# A case of pediatric primary osteolytic extradural and complicated hydatid cyst revealed by a skull vault swelling

**DOI:** 10.1007/s00381-023-05999-w

**Published:** 2023-05-27

**Authors:** Mehdi Borni, Souhir Abdelmouleh, Marouen Taallah, Hela Blibeche, Ali Ayadi, Mohamed Zaher Boudawara

**Affiliations:** 1 Department of Neurosurgery, UHC Habib Bourguiba, Sfax, Tunisia; 2 Department of Neurology, UHC Habib Bourguiba, Sfax, Tunisia; 3 Department of Parasitology and Mycology, UHC Habib Bourguiba, Sfax, Tunisia

**Keywords:** Extradural, Echinococcosis, Skull vault swelling, MRI, Surgery

## Abstract

Hydatidosis is a parasitic infestation whose etiological agent is the larva of the cestode *Echinococcus granulosus*. It is a zoonosis, and the human being behaves as an accidental intermediate host in the parasitic cycle with pediatric predominance. The most frequent clinical presentation is hepatic, followed by pulmonary, with cerebral hydatidosis being extremely rare. Imaging is characteristic, generally dealing with single cystic lesion, usually unilocular and less frequently multilocular, located mainly intraaxially. Extradural hydatid cyst, whether primary or secondary, remains very rare or even exceptional. The primary disease remains extremely rare, and its clinical picture is related to the number, size, and location of the lesions. Infection within these cerebral hydatid cysts remains an extremely rare occurrence, and only few cases were reported previously in the literature. The authors report the nosological review of the clinical, imaging, surgical, and histopathological records of a pediatric primary osteolytic extradural and complicated hydatid cyst in a 5-year-old North African male patient coming from a rural area who presented for progressive onset of a painless left parieto-occipital soft swelling without any neurological disorder with good outcomes after surgery. The authors report this case due the fact that it had not been documented before in the pediatric population and to the success of the specialized treatment.

## Introduction

Hydatidosis is a parasitic infestation whose etiological agent is the larva of the cestode *Echinococcus granulosus* due to the ingestion of eggs in the excretions of infected carnivores, raw vegetables or fruits, and contaminated water [1, 2]. It is a zoonosis and the human being behaves as an accidental intermediate host in the parasitic cycle [1, 3]. The most frequent clinical presentation is hepatic (50–77%), followed by pulmonary (8.5–43%), with cerebral hydatidosis being extremely rare (2%) [4, 5]. Seventy five percent occurs in children [1], and male patients are more affected [4]. The mass effect exerted by the cyst on the different brain structures is primarily responsible for the clinical manifestations of the disease, which will vary according to the cyst’s location. Imaging characteristic, generally dealing with single cystic lesion, usually unilocular and less frequently multilocular, located mainly intraaxially. Extradural hydatid cyst, whether primary or secondary, remains very rare or even exceptional [4]. Infection within these cerebral hydatid cysts remains an extremely rare occurrence and only few cases have been reported previously in the literature [6–11].

Here, the authors report the nosological review of the clinical, imaging, surgical, and histopathological records of an uncommon primary osteolytic pediatric extradural complicated hydatid cyst in a 5-year-old North African male patient coming from a rural area who presented for progressive onset of a painless left parieto-occipital soft swelling without any neurological disorder with good outcomes after surgery.

The authors report this case due the fact that it had not been documented before in the pediatric population and to the success of the specialized treatment.

## Case report

**Fig. 1:**
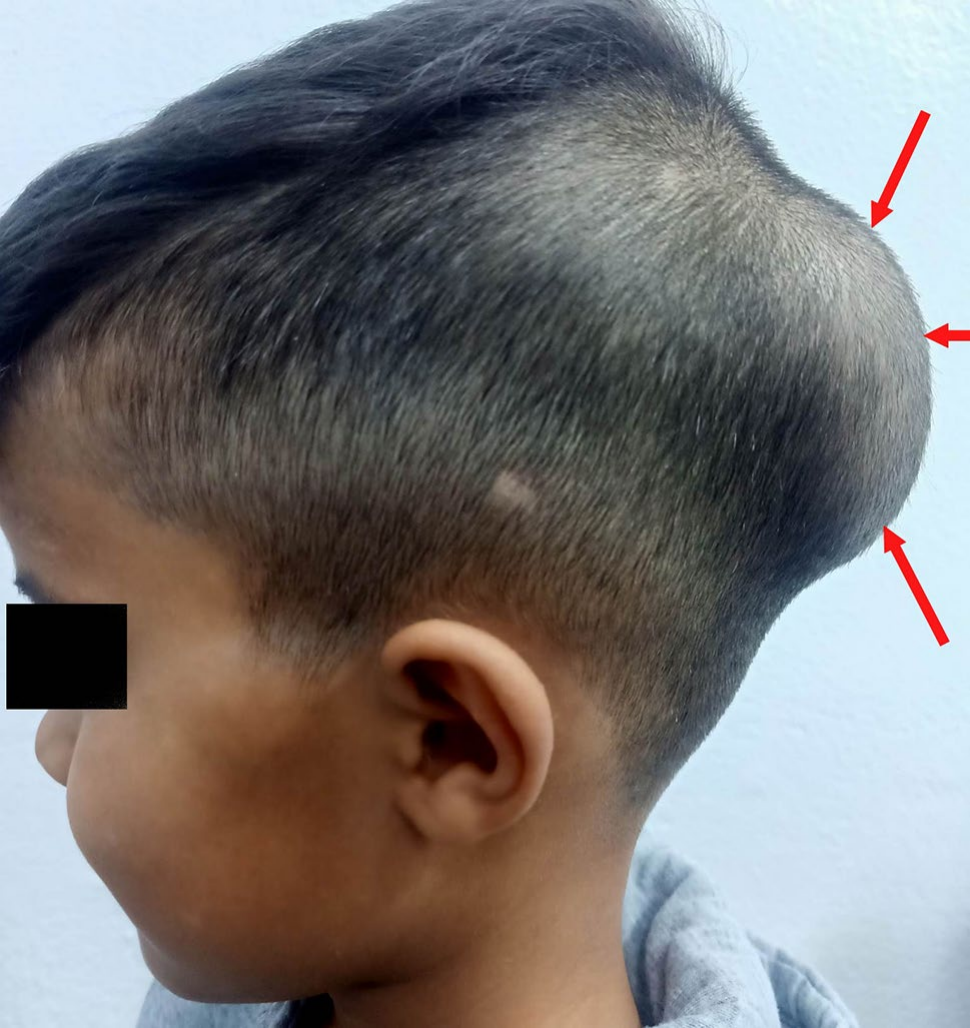
Photograph of the patient showing a parietal swelling (red arrows) covered with normal intact skin without any local redness or inflammatory signs

A 5-year-old preschooler North African male patient from a rural area where cattle are raised and with the habit of feeding pets (dogs) with their entrails was referred from his local dispensary to our department of neurosurgery for progressive onset of a painless left parieto-occipital soft swelling. His mother first noticed the swelling 2 months ago. Upon examination, the boy was alert, awake, with spontaneous ventilation and stable vital signs, as well as a normal cardiac and pulmonary profile, without any neurological disorder. The rest of his physical assessment showed a soft swelling of about 4 cm in size rising in front of his parieto-occipital bone (Fig. 1). This mass was barely mobile, painless, and covered with normal intact skin without any local redness or inflammatory signs. There was no history of night sweats, weight loss, fever, or any episodes of previous head trauma.

**Fig. 2:**
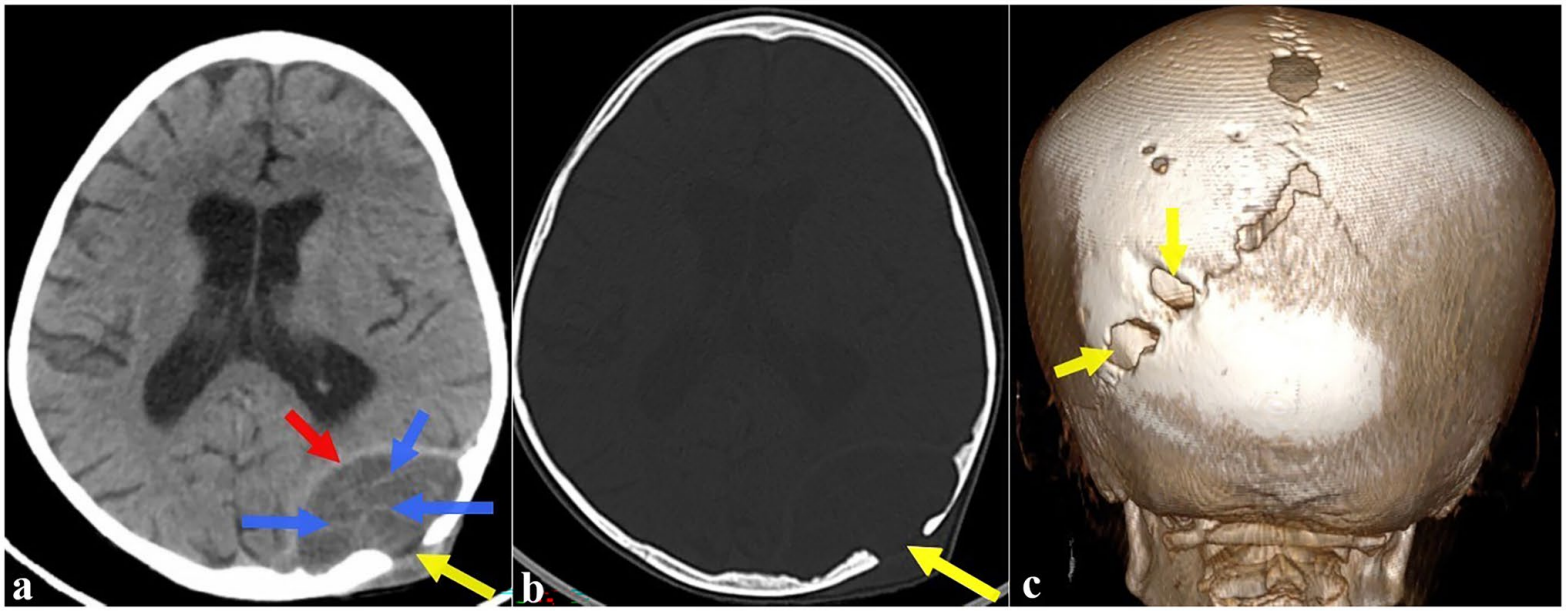
Axial non enhanced brain CT scan in parenchymal window (**a**), bone window (**b**) with 3 dimensional reconstruction (**c**) showing a posterior extra parenchymal left parietal cystic mass (**a**, red arrow) communicating with the subcutaneous tissues through bone defects (**a**, **b**, **c**, yellow arrows) containing membranes (**a**, blue arrows)

**Fig. 3:**
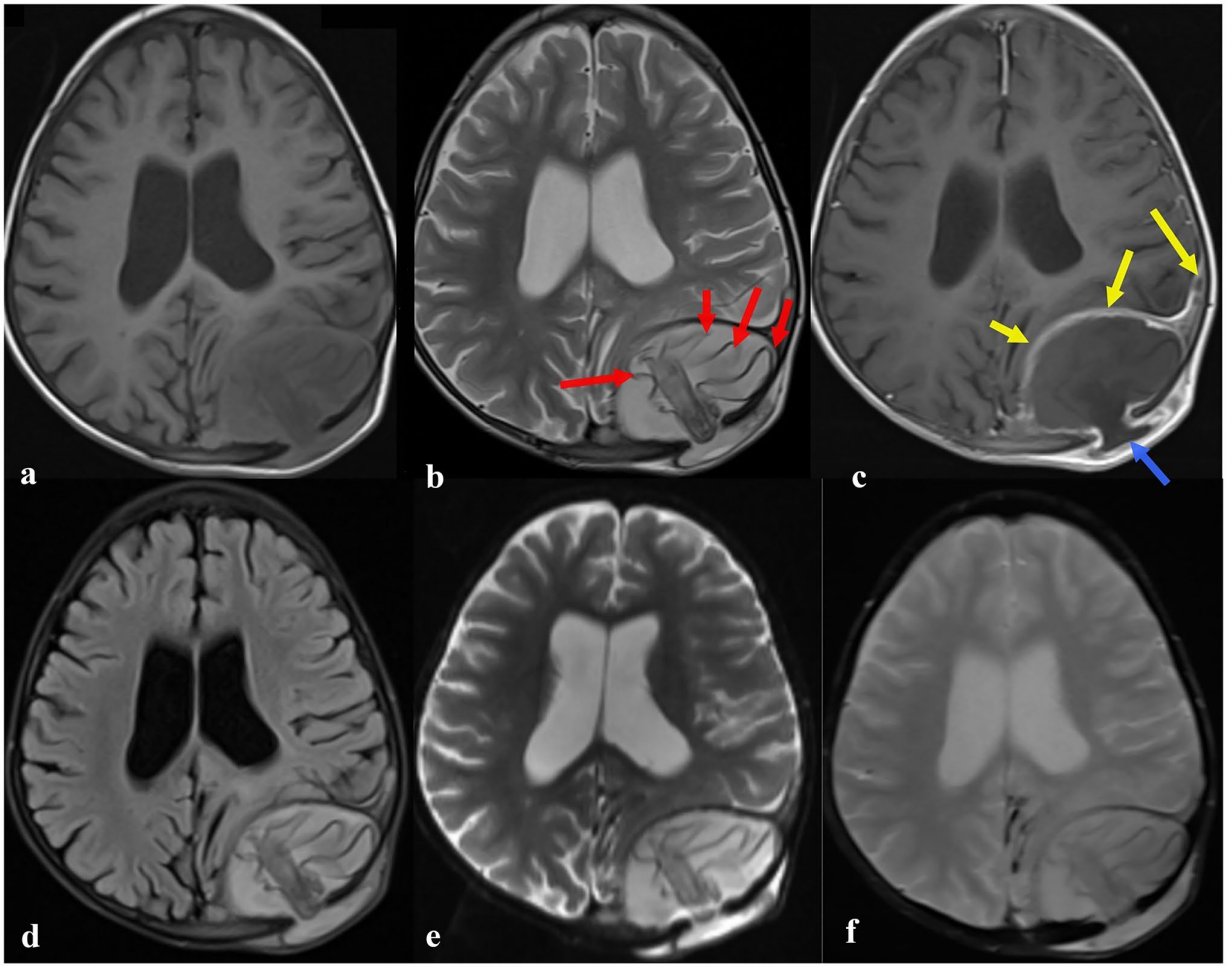
Axial brain MRI showing a well-limited extra-axial lesion of heterogeneous liquid signal, in hyposignal on T1-weighted sequences (**a**) and hypersignal on T2-weighted sequences (**b**) with floating membranes (**b**, red arrows) and a wall in hyposignal on T2-weighted sequence. Note the peripheral rim enhancement as well as of the adjacent dura mater after gadolinium chelates injection (**c**, yellow arrows). Note the absence of edema surrounding the cyst on the T2-fluid attenuated inversion recovery sequences (FLAIR) (**d**) as well as the absence of any abnormal signal on diffusion weighted sequences (**e**) and gradient echo sequences (**f**)

The brain computed tomography (CT) scan (Fig. 2) performed at admission showed a posterior extra parenchymal left parietal cystic mass measuring 64 × 70 mm in diameter communicating with the subcutaneous tissues through bone defects containing membranes. Further investigation by magnetic resonance imaging (MRI) (Fig. 3) confirmed the well-limited extra-axial lesion of heterogeneous liquid signal, in hyosignal on T1-weighted sequences and in hypersignal on T2-weighted sequences which is the seat of linear floating membranes with a wall in hyposignal on the weighted sequences in T2. After the gadolinium chelates injection, there is peripheral rim enhancement as well as of the adjacent dura mater without cruoric endoluminal thrombus of the superior sagittal venous sinus. The whole is associated with multiple foci of parietal bone lysis with an extension towards the subcutaneous tissues measuring 48 × 18 mm in diameter. All these radiological features suggested brain hydatidosis.

**Fig. 4:**
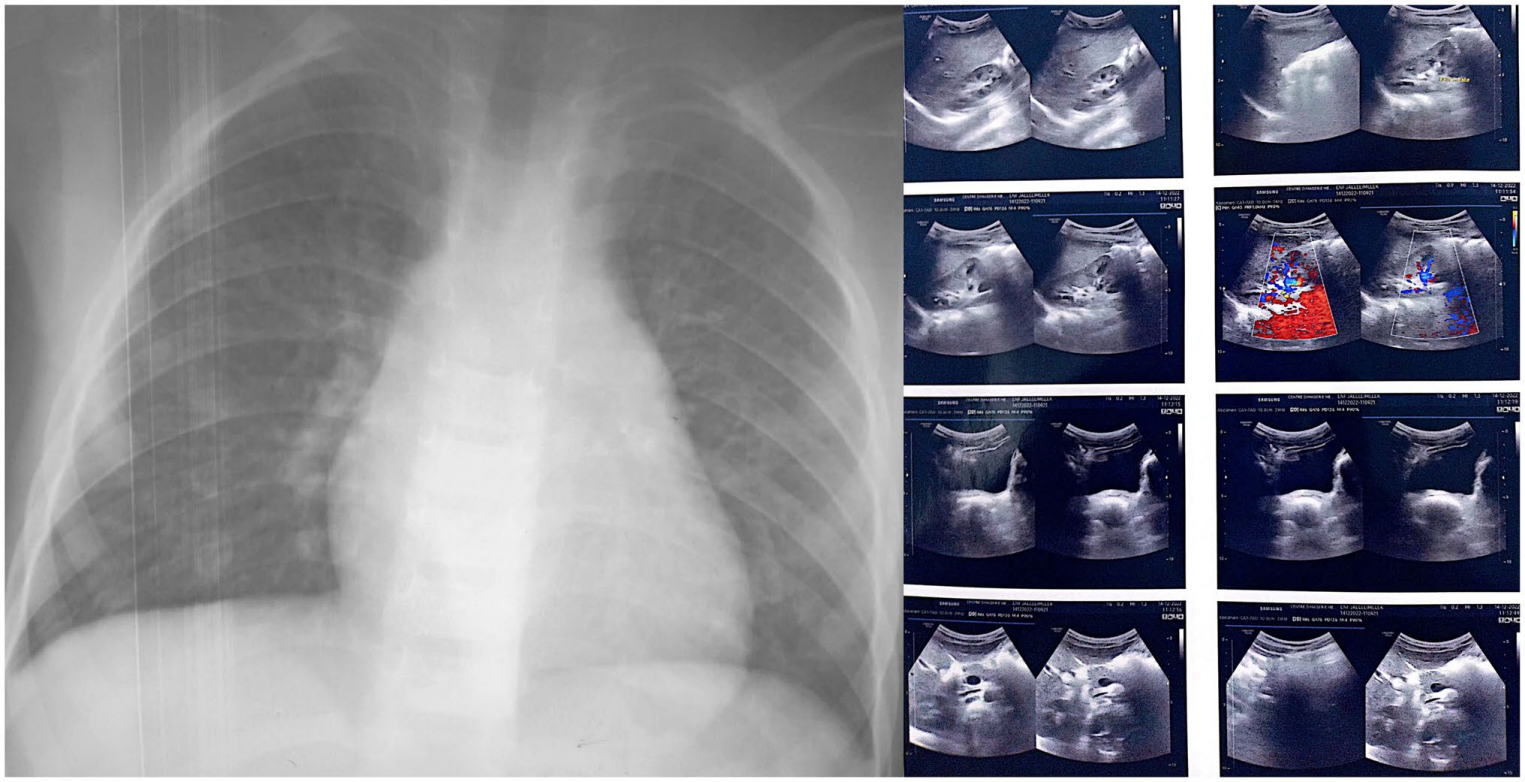
Posteroanterior chest x-ray (left image) and abdominal ultrasound (right image) showing no abnormalities

Within the framework of the investigation in search of other pulmonary or hepatic localizations of the hydatid pathology, the posteroanterior chest x-ray did not show any radiopaque images or cavitated lesion. The abdominal ultrasound was also without abnormalities (Fig. 4). The complete blood count, the serum electrolytes test, and hemostasis assessment revealed no abnormalities. Serological test trying to assess hydatidosis revealed negativity for the crude larval-antigen enzyme-linked immunosorbent assay (ELISA) and the presence of the 7-kDa band by the classic western blot technique confirming the serological diagnosis of hydatidosis.

Our patient received albendazole orally at a dose of 15 mg/kg of his body weight daily in 2 divided doses in order to reduce the risk of recurrence and facilitate surgery by reducing intracystic pressure.

**Fig. 5:**
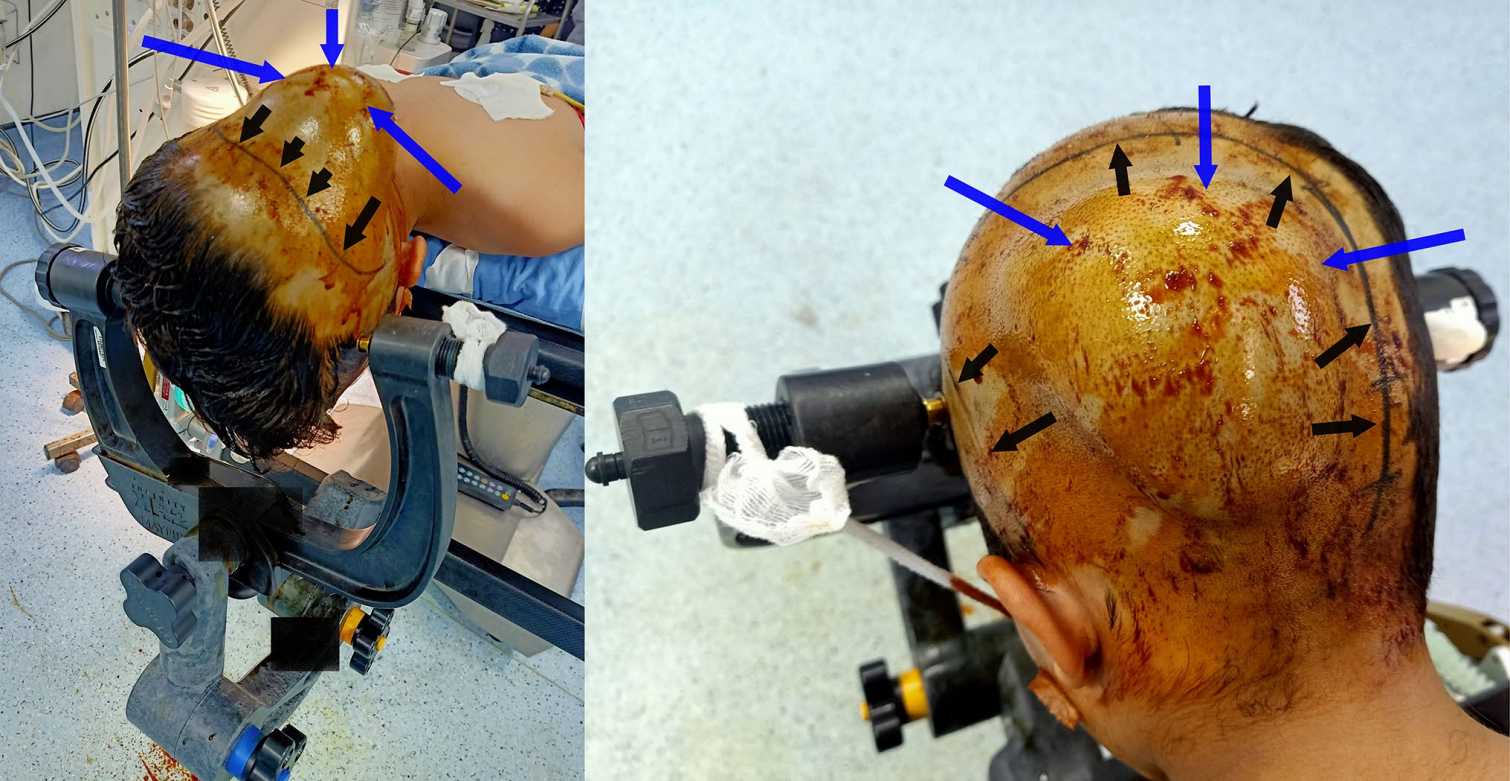
Photograph of the patient put in a prone position with head immobilized in Mayfield^®^ three-pin skull clamp. The skin incision (black line, black arrows) made in “c” shaped centered on the swelling (bleu arrows)

**Fig. 6:**
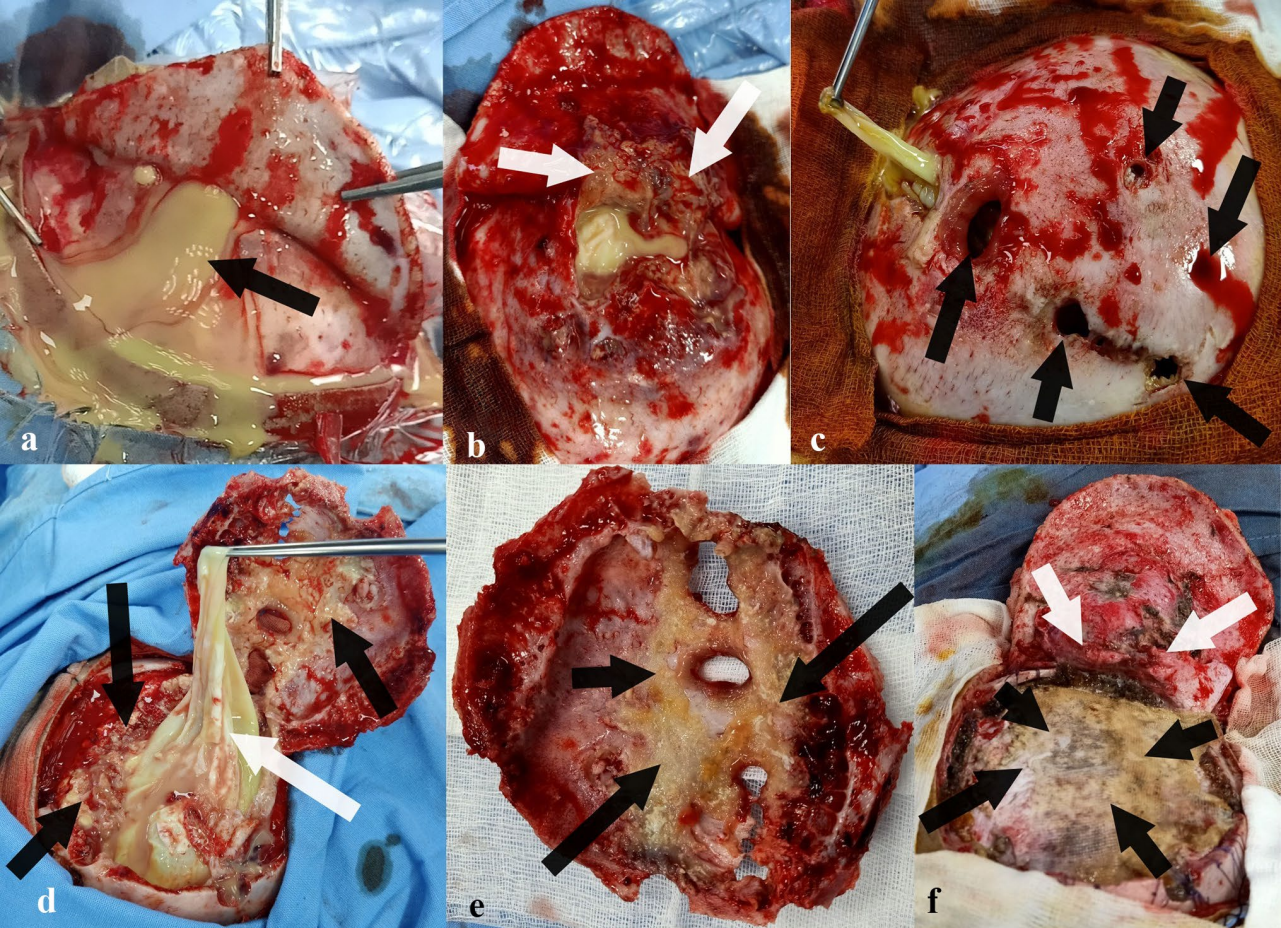
Peroperative photographs showing an abundant discharge of greenish-yellow pus upon dissection and detachment of various subcutaneous tissues (**a**, black arrow). The galea aponeurotica is thickened and infiltrated (**b**, white arrows) with various bone defects (**c**, black arrows). After bone flap removal (**e**), the infected material (**d** and **e,** black arrows) and a thick yellowish membrane were visualized (**d**, white arrow). The operative site was carefully cleaned and freed of various pus debris allowing visualization of healthy dura mater (**f**, black arrows) as well as the infected part of the galea aponeurotica which was radically resected (**f**, white arrows)

After an informed consent from his legal guardian, the boy underwent surgery. In the operating room, he was put in a prone position with his head immobilized in the Mayfield^®^ three-pin skull clamp (Fig. 5). The skin incision was “c” shaped with an inferior hinge centered on the swelling and having a broad base to avoid compromising its vascularity (Fig. 5). Upon dissection and detachment of the various subcutaneous tissues, an abundant discharge of greenish-yellow pus occurred which was softly sucked (Fig. 6). The galea aponeurotica also appeared thickened and infiltrated and its groaning exposed the vault as well as the various bone defects already visualized on the CT scan (Fig. 6). After the bone flap removal, the infected brownish material and a thick yellowish membrane were visualized (Fig. 6). The operative site was carefully cleaned and freed of various pus debris showing the healthy dura (Fig. 6). The operative site was profusely irrigated with 3% hypertonic saline for 20 min intraoperatively. The already infected and perforated bone flap was not put back in place and was sent, therefore, for histological study. The infected brownish material and the thick yellowish membrane were also sent for laboratory examination. Closure of the different subcutaneous tissues was designed according to the usual technique after resection of the infected part of the galea aponeurotica. The immediate postoperative course was uneventful without delayed awakening from anesthesia. No adverse reactions were verified during surgery or after the event. Our patient was put under empiric antibiotic therapy based on cefotaxime (200 mg/Kg/day in 4 divided doses), vancomycin (80 mg/day), and metronidazole (15 mg/Kg/day in 3 divided doses).

**Fig. 7:**
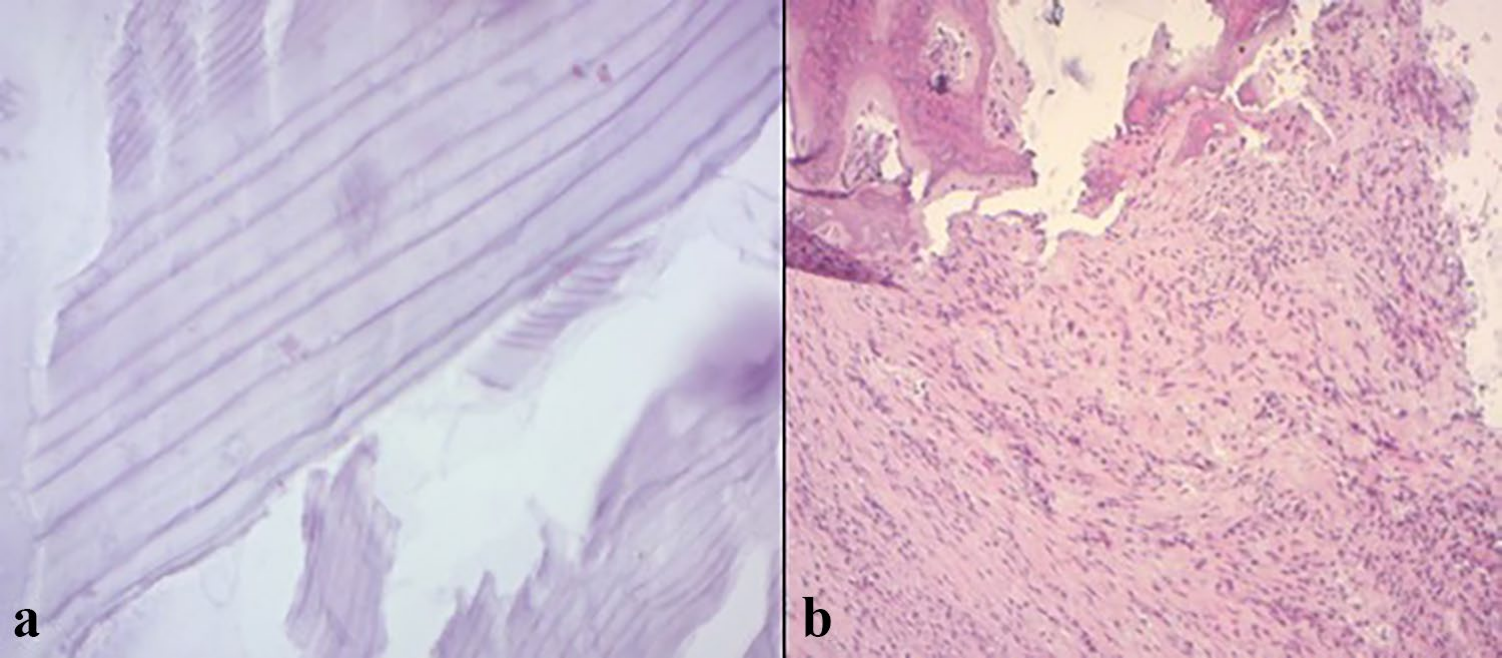
Histopathological examination showing the thick cuticular membrane, anhist, with characteristic lamellar appearance (**a**; HE × 200). The pericyst was formed by fibro-inflammatory tissue, adhering to the cranial bony trabeculae (**b**; HE × 200)

The histopathological examination (Fig. 7) of various samples revealed a whitish membrane that corresponded to a thick, anhistic, eosonophilic, and lamellar cuticular material. The brownish fragments were formed by a fibrous and congestive tissue, comprising an abundant and polymorphic granulation tissue. The cyst was adherent to the bone tissue and dissociated by various inflammatory changes. All these features are in favor of infected hydatid cyst. The bacterial culture of pus in various bacteriological culture media was negative.

**Fig. 8:**
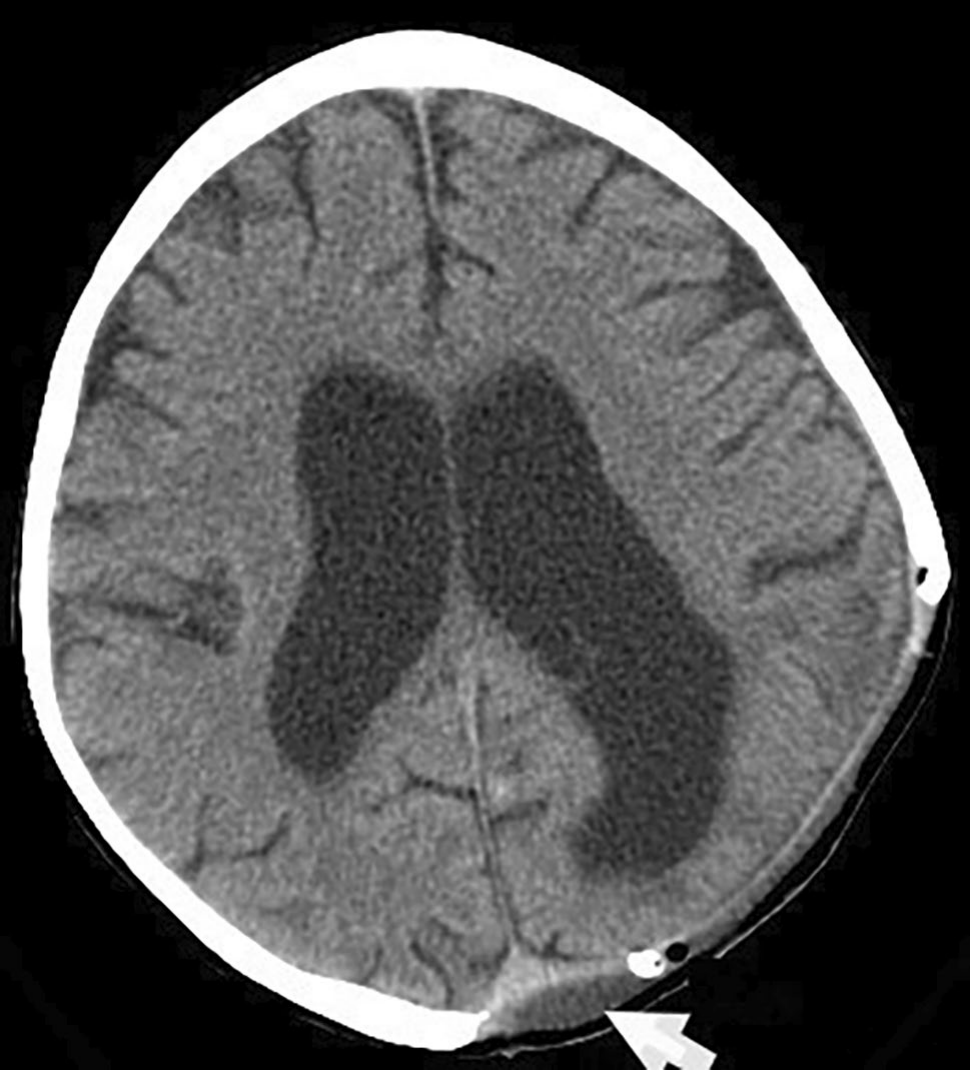
Axial postoperative non enhanced CT scan in parenchymal window performed 2 days postoperatively showing a subcutaneous collection next to the surgical site (white arrow) with an air bubble. Note the disappearance of the extra parenchymal cystic mass and its membranes

The brain CT scan performed 2 days postoperatively (Fig. 8) showed the disappearance of the extra parenchymal cystic mass as well as its membranes. The subcutaneous postoperative collection next to the surgical site contained with an air bubble collapsed by closed Redon suction drainage which was removed on day 5 postoperatively. The patient was put under clinical and biological monitoring and remains stable. He was discharged 60 days later at the end of his antibiotic therapy course and scheduled for cranioplasty, in good general and neurological conditions, being monitored on an outpatient basis; managing to remain asymptomatic. During this period, he received complementary medical treatment with cysticidal antiparasitics (albendazole 400 mg orally, every 8 h, for 2 weeks and in 2 cycles).

## Discussion

Hydatidosis is the infestation produced by the *Echinococcus granulosus* parasite whose final hosts are canine animals. Sheep, cattle, and goats intervene as intermediate hosts. Man is an accidental intermediate host by ingesting food contaminated with fecal matter containing parasite eggs. The larvae released in the human intestinal lumen invade the bloodstream and spread to different organs [1, 3]. Our patient originated from a rural area where cattle are raised and with the habit of feeding pets (dogs) with their entrails which may explains its logical infestation by the cestode. The diagnosis of hydatidosis is based on epidemiological data, clinical manifestations, and complementary imaging methods. Serological tests can help the diagnosis [12]. The frequency of cerebral hydatidosis varies between 0.5 and 3%, as described in the studies by Turgut [13], Kovoor [14], and Onal [15]. Cerebral localization affects children in up to 75% of cases. Some authors report that male children are more affected [4, 16, 17], others such as Limaiem et al. [18] did not find significant differences.

Hydatidosis may present in two ways depending on the mechanism by which the lesions occur in the central nervous system: primary or secondary [19]. The primary form corresponds to the presentation as a single cyst located in the territory irrigated by the middle meningeal artery. Multiple primary form is known to be with an anecdotal presentation [20], having reported only 20 cases in the world in related searches by PUBMED, SCOPUS, EBSCO, BIREME, and Google Scholar to date [21]. In the secondary form, there are multiple cysts of any location generally associated with involvement of other organs leading to embolization of pup-vesicles to even the brain [19]. Our patient presented with the primary form as his chest x-ray as well as his abdominal ultrasound did not reveal any abnormalities.

The growth of the hydatid cyst is usually slow, with a growth rate that ranges from 1.5 cm/year according to some reports [22, 23]. The mass effect exerted by the cyst on the different brain structures is primarily responsible for the clinical manifestations of the disease, which will vary according to the cyst’s location. Bütke et al. [4] reported headache (83%), papilledema (77%), vomiting (66%), hemiparesis (38%), seizures (33%), visual disturbances (27%), ataxia (11%), and facial paralysis (5%). Duishanbai et al. [24] reported 30 cases of cerebral hydatidosis, where the forms of clinical presentation were headache and vomiting (87%) and hemiparesis (30%). Our patient did not present any of these neurological symptoms which makes his case rare despite the large size of his cyst (64 × 70 mm in diameter). The calvarial swelling with which our patient presented is therefore a rarity in itself. To our knowledge, very few cases have been reported previously in the literature in adult patients [25–28].

The mass effect of the lesion determines also some of the main complications, such as obstructive hydrocephalus, midline shift, and brain herniation [4, 29, 30]. These latter complications were fortunately absent in our patient.

Infection within cerebral hydatid cysts remains an extremely rare occurrence and only few cases have been reported previously in the literature [6–11]. Moreover, suppuration in skull-eroding hydatidosis as reported in our case remains also extremely rare [31]. The exact mechanism of this suppuration is still poorly understood. The source of infection is usually coexisting bacterial infection with contiguous contamination or secondary hematogenous spread [32]. Our patient had no stigma of infection including his blood cultures which turned out to be negative.

Findings from the imaging point of view are characteristic, generally dealing with single cystic lesions, usually unilocular and less frequently multilocular, located mainly intraaxially and more frequently hemispherical in the vascular territory of the middle meningeal artery due to arrival via hematogenous parasite. Reviewing the literature database, we noted that the incidence of extradural hydatid cysts is quite low. We found only 12 cases that developed either directly from the skull, from the extradural vessels, or by its inoculation through an intact dura mater [17]. At the same time, only one case has been published so far of multiple hydatid cysts with extradural localization [33, 34].

The lesion is usually well defined, with isodense and isointense content compared to the cerebrospinal fluid, without perilesional edema and minimal or absent contrast uptake in its periphery [4, 29, 35]. In our patient, the lesion was fully extra-axial with heterogeneous liquid signal. After the gadolinium chelates injection, there is a peripheral rim enhancement as well as of the adjacent dura mater. The whole was associated with multiple foci of parietal bone lysis with an extension towards the subcutaneous tissues which is totally discordant with the literature where no others previous case was reported showing bone lysis with extra-axial hydatid cyst.

The lack of surrounding edema and marked mass effect make it easy to distinguish hydatid cyst from abscess or cystic tumor; likewise, arachnoid cysts, porencephalic cysts, and epidermoid tumors should be taken into consideration in the differential diagnosis [23]. There are characteristics that differentiate them: arachnoid cysts are not spherical, porencephalic cysts are generally connected to the ventricular system and are also not completely surrounded by brain tissue, cystic tumors usually have soft tissue components that enhance after contrast injection, and abscesses typically demonstrate peripheral contrast enhancement and perilesional edema. When the site is atypical, such as an infratentorial lesion, the differential diagnosis is made with neuroenteric cysts [36].

Regarding echocardiography, lung x-rays, abdominal ultrasound, or immunology studies, the results are contradictory or erratic. If right-left cardiac communications were found, we could assume the disease as secondary due to the possibility of parasitic metastasis and its subsequent development in the brain. Although it has not been proven that its presence is an indisputably associated risk [21]. Thus, in our case, no extracerebral image has been useful to strengthen the diagnosis of a primary site of hydatidosis, which satisfies the denomination of primary brain disease.

Serology by ELISA technique is not very useful in cerebral location, less than 10% of cases are positive. The indirect hemagglutination technique has a sensitivity of 80% in liver localization and 65% in lung lesions [12]. There are usually no alterations in the usual hematological studies and their finding is non-specific [1, 3].

The treatment of choice is surgical resection of the cyst using the Dowling-Orlando technique [1, 13], which consists of total excision of the cyst via performing a wide craniotomy with subsequent opening of the adventitia and irrigation of saline solution between the brain parenchyma and the cyst that allow the intact exit of the germinal layer and its contents by gravity, then tilt the patient’s head leading to the delivery of the cyst. In the case we present, we could not perform this technique as the cyst was not intact and has communication with the subcutaneous tissues via the bone lysis. Among the cysts with intraosseous origin and with bone erosion towards the extradural space, only one case has been published with intact ablation of the cyst without its rupture. Thus, we can conclude that it is a real challenge to remove the intact cyst in case of bone invasion [6, 37].

Hypertonic saline solution is an alternative way to prevent recurrences or secondary hydatidosis [38]. Spektor et al. performed 3% hypertonic saline irrigation for the treatment of an extradural intravertebral hydatid cyst. Their patient fully recovered from his quadriplegia [39]. Hypertonic saline solution induces coagulant necrosis in different tissues. It increases the electrical conductivity of the tissue and raises its temperature. In our case, we performed profuse irrigation of the operative site with 3% hypertonic saline for 20 min intraoperatively to prevent any recurrence.

Secondary recurrence to intraoperative rupture, meanwhile, reaches 40.7% [18, 40, 41]. In Turgut’s case series, 5 of 137 cases of intracranial hydatidosis, definitive treatment consisted of surgical resection in 85% of cases, with accidental rupture of the cyst occurring in 25% [13]. In the case series by Tuzun et al. [42], which included 25 children who underwent surgical resection of cerebral hydatid cysts, 12% presented cyst rupture during surgery, without development of anaphylaxis. Other complications that were observed were the following: pneumocephalus (12%), subdural effusion (20%), hemorrhage, and epidural hematoma in less than 10% of cases. Our patient did not have any of these complications.

The antiparasitic of choice in hydatidosis is albendazole. Its use is controversial and variable according to world experience. Prior to surgery, the risk of disease dissemination would be reduced, as would the release of viable scolices and hydatid fluid that could cause fatal anaphylactic reactions. On the other hand, the treatment could thin the cystic wall with the consequent risk of rupture during the procedure [13, 43]. In the experience of Turgut [13] in Turkey, albendazole or mebendazole was used in 60% of the cases. It was indicated due to accidental rupture of the cyst or due to the impossibility of surgical resolution due to multiple cysts. Some authors recommend starting treatment with albendazole 10 mg/kg/day orally prior to surgery and continuing it for at least 3 months afterwards [44]. Our patient received albendazole orally at a dose of 15 mg/kg of his body weight daily in 2 divided doses in order to reduce the risk of recurrence and facilitate surgery by reducing intracystic pressure. Another group of experts recommend the use of albendazole only in those patients with multiple intracranial cysts or in another location, residual post-surgical cysts, or in cases of intra-surgical rupture [24].

To our knowledge, our patient is the first case of pediatric complicated primary cerebral hydatidosis presenting in an uncommon way by a scalp swelling with atypical radiological features including extradural location, infection, and bone lysis. Furthermore, surgery was not performed according to Dowling-Orlando’s technique due to cyst’s location and presentation with simple postoperative course.

## Conclusion

Cerebral hydatidosis is a disease of parasitic origin and extremely rare presentation, the pathophysiology of which is still poorly understood in view of its biological and immunological behavior, but which requires a high clinical-epidemiological index of suspicion and consistent neuroimaging evidence to the diagnostic approach. Surgical resection of cysts is undoubtedly the treatment of choice. It is still necessary to gather more information related to its nosology, as well as to strengthen epidemiological control strategies for primary prevention purposes in different regions and countries.

## Data Availability

Mehdi Borni was responsible for the work and the conduct of the study, had access to the data, and controlled the decision to publish.
